# Understanding of Crucial Factors for Improving the Energy Density of Lithium-Sulfur Pouch Cells

**DOI:** 10.3389/fchem.2022.888750

**Published:** 2022-05-02

**Authors:** Olatz Leonet, Álvaro Doñoro, Ana Fernández-Barquín, Andriy Kvasha, Idoia Urdampilleta, J. Alberto Blázquez

**Affiliations:** CIDETEC, Basque Research and Technology Alliance (BRTA), Donostia-San Sebastián, Spain

**Keywords:** lithium-sulfur battery, sulfur loading density, cell balancing, pouch cell performance, electrolyte volume, high energy density

## Abstract

Rechargeable lithium−sulfur (Li−S) batteries are the most promising next-generation energy storage system owing to their high energy density and low cost. Despite the increasing number of publications on the Li-S technology, the number of studies on real prototype cells is rather low. Furthermore, novel concepts developed using small lab cells cannot simply be transferred to high-energy cell prototypes due to the fundamental differences. The electrolyte and lithium anode excess used in small lab cells is known to have a huge impact on the cycle life, capacity, and rate capability of the Li-S system. This work analyses the performance of pouch cell prototypes demonstrating the potential and hurdles of the technology. The impact of electrolyte variations and the sulfur cathode loading are studied. The energy density of Li-S pouch cell is improved up to 436 Wh kg^−1^ by a combination of different approaches related to cell manufacturing, sulfur cathode optimization, and electrolyte amount adjustment.

## Introduction

Lithium-sulfur (Li-S) batteries have attracted the attention of researchers in recent years for their numerous benefits (Ould Ely et al., 2018). Namely, their high theoretical specific capacity (1,672 mAh g^−1^) and high theoretical specific energy density (2,567 Wh kg^−1^) largely exceed that of conventional lithium-metal batteries based on intercalation cathodes, where the theoretical specific energy is around 1,000 Wh kg^−1^ (Abraham, 2019). Consequently, Li-S batteries are one of the favorite candidates for the forthcoming generation of energy storage technologies. Indeed, elemental sulfur, the active material of Li-S batteries, has the potential to meet the requirements of high theoretical capacity, abundance, and environmental friendliness (Nazar et al., 2014).

However, the novel Li-S technology has several limitations: 1) the low electrical conductivity of sulfur (5·10^−30^ S cm^−1^), and 2) the dissolution of intermediate lithium polysulfides (LiPSs) from the cathode, Li_2_S_n_ (2 ≤ n ≤ 8), that results in shuttle reactions, known as “shuttle-effect” (Manthiram et al., 2013). As a result, Li-S batteries typically show poor Coulombic efficiency, fast discharge capacity degradation, and a severe self-discharge (Yan et al., 2016). Furthermore, issues are related to the growth of lithium dendrites at the anode and the corrosion of the anode caused by detrimental reactions between porous lithium metal deposits and liquid electrolytes, but those have not been deeply investigated so far (Chen X. et al., 2017; Cheng et al., 2018; Liu Y. et al., 2021).

In order to address the mentioned issues at the cathode side, elemental sulfur is usually confined in micro-nanosized carbon matrixes (i.e., electronically conductive material), which enhances the sulfur utilization and traps dissolved LiPSs (Xu et al., 2018; Doñoro et al., 2019; García et al., 2020). This minimizes the shuttle reaction and improves the cycling performance. However, the required amount of carbon support should be significant, which, in turn, greatly reduces the Li-S energy density and complicates the preparation and upscale of homogeneous sulfur-carbon composite cathodes with a reasonable sulfur loading per unit area (e.g. 3.0–5.0 mg_s_ cm^−2^) (Borchardt et al., 2016; Peng et al., 2017).

Most of the essential breakthroughs on Li-S batteries aimed to overcome the abovementioned problems individually, were achieved at the laboratory scale, i.e. in coin-cell configurations with favorable conditions, such as high negative to positive (N/P) electrode ratio (N/*p* > 25), areal sulfur loadings (ASL) below 2 mg_s_ cm^−2^, and high electrolyte volume to capacity (E/C) ratio (E/C > 10 µl mAh^−1^) (Gomez et al., 2018; Zhao et al., 2020b; Shi et al., 2020). By doing so, researchers have learned that the electrochemical performance of Li-S coin cells can be significantly improved especially when excessive electrolyte amount is used. In turn, it notoriously reduces the energy density at the cell level. Moreover, it should be also noted that studies conducted at the coin-cell level typically show important differences with regard to the delivered specific charge/discharge capacities or the cell cycling stabilities, even if the electrode materials and the cell fabrication protocol remain similar. In addition to that, the reported results usually exhibit a major contrast between the coin and pouch formats, which hinders the scalability and further commercialization of the Li-S batteries (Zhu et al., 2019). Therefore, evaluating Li-S batteries at the pouch cell level is essential to obtain meaningful insights into their practical implementation (Chung et al., 2018; Yang et al., 2018).

**FIGURE 1:**
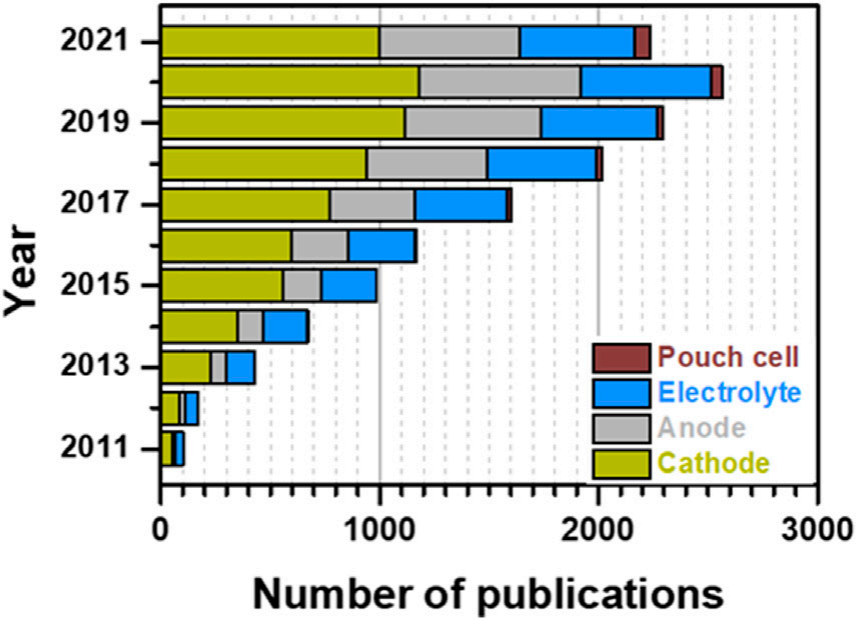
Publication trend (from 2011 to 2021) of Li-S research reported in the literature. Data were obtained from the Web of Science in December 2021. NOTE: Literature survey was performed according to each defined topic. Since most publications cover several given subjects, the real amount of total publications per year, concerning Li-S batteries as a whole topic, might slightly vary from the data reported for each year in this figure.

**FIGURE 2:**
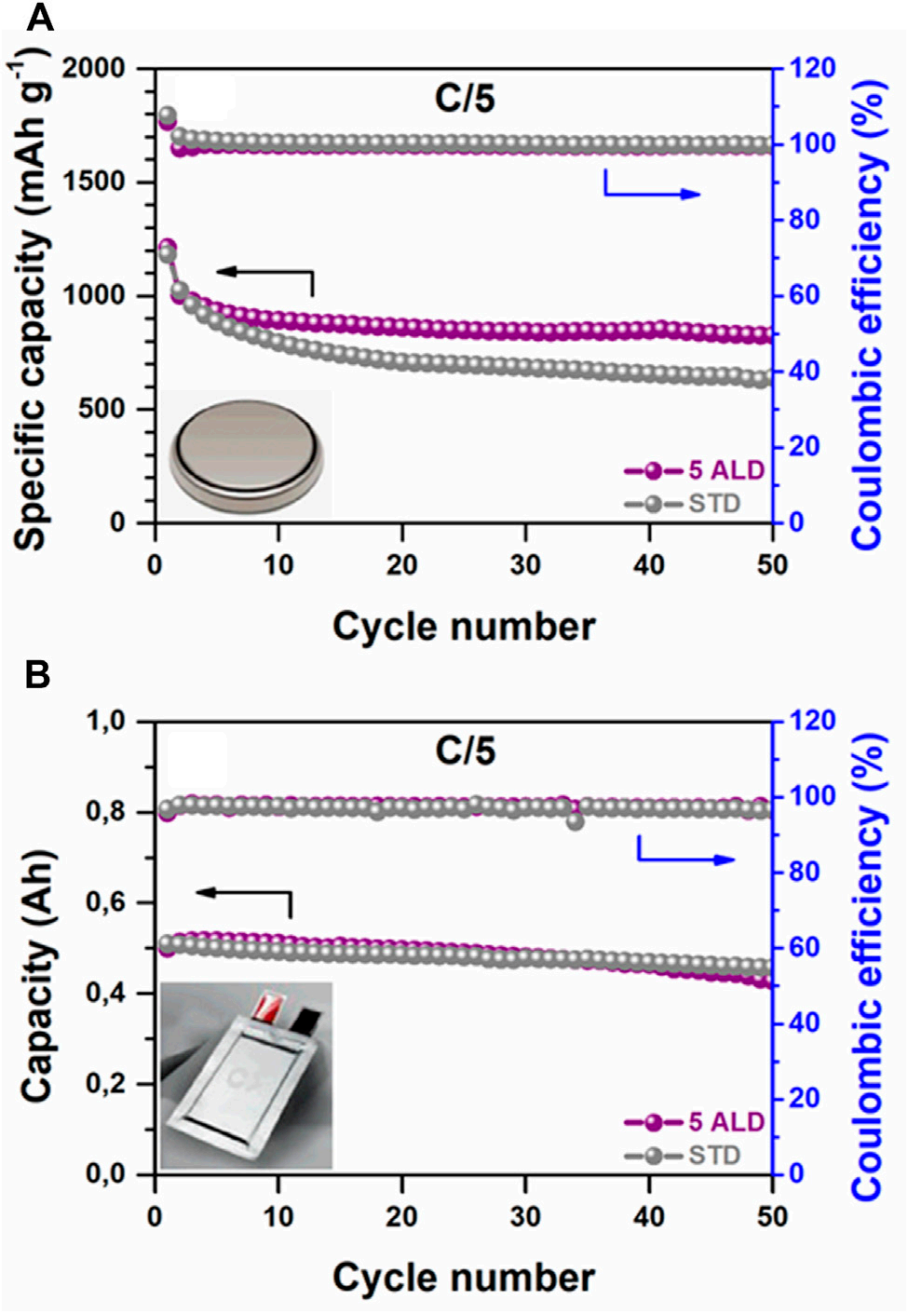
Electrochemical performance of rechargeable standard (STD) and FBR-ALD coated (5 ALD) sulfur cathodes in Li-S batteries was measured at 25°C. Galvanostatic long cycling performance at C/10 in **(A)** coin cell and **(B)** pouch cell format.

Although the characterization of multi-layered pouch Li-S cells is not commonly reported in the scientific literature, several studies that described interesting results were published over the last decade (Figure 1) (Chung and Manthiram, 2018; Ye et al., 2022). Noteworthy, most of the reported Li-S pouch cells were fabricated in a one-layer format, due to the challenging complexity of the multilayered pouch cells assembly process. In all the cases, the inactive components played a key role by adding a “dead weight” to the Li-S battery, thus decreasing the overall energy density at the pouch cell level. Therefore, the optimization of the sulfur electrode appears as one of the most relevant issues to be considered, because increasing the ASL and implementing an optimized balance of the sulfur/carbon ratio could lead to higher energy densities. For example, in our previous work, a fluidized bed reactor (FBR) was used to coat alumina (Al_2_O_3_) by atomic layer deposition (ALD) on sulfur-carbon (S/C) composite particles (Azaceta et al., 2020). This cost-effective, easily scalable, and highly efficient approach improved the cohesion between the positive electrode components by agglomerating and interconnecting the particles of the S/C composite, which allowed increasing the ASL from 1.8 mg_s_ cm^−2^ in the reference cathode (STD) to 3.6 mg_s_ cm^−2^ in the cathode based on FBR-ALD Al_2_O_3_-coated S/C particles. Moreover, for a fixed ASL, boosted specific charge/discharge capacities were observed at the coin cell level, where the modified cathode outperformed the STD cathode (Figure 2A) (Azaceta et al., 2020). However, results at the pouch cell level somehow differed from the coin cell results, since the electrochemical performance of STD and modified cathode-based pouch cells exhibited minor differences under lean electrolyte conditions (Figure 2B). These results reveal that the other factors should be also studied toward the development of high energy-density Li-S batteries, which are in good agreement with the literature (Hagen et al., 2015; Chung et al., 2018).

From all additional factors, the electrolyte also represents a critical issue in Li-S pouch-cells due to its relatively high weight fraction (>45 wt%), which greatly exceeds its fraction in conventional lithium-ion (Li-ion) batteries (∼20 wt%) (Liu T. et al., 2021). This fact is mainly caused by the lower weight contribution of the active material due to the high specific capacity of sulfur, where even highly-loaded cathodes in Li-S batteries (>5 mg_s_ cm^−2^) cannot compete with the corresponding cathodes of Li-ion batteries (areal loading of >20 mg cm^−2^). Therefore, reducing the electrolyte amount in Li-S pouch cells represents an effective approach to improving the energy density (Cheng et al., 2017; Chen et al., 2019a). In order to match and exceed the energy density of Li-ion batteries, previous studies have reported key E/C values for the most standard Li-S electrolyte solution, based on 1 M lithium bis(trifluoromethanesulfonyl)imide (LiTFSI) and 2 wt% of lithium nitrate (LiNO_3_) in a mixture of 1,2-dimethoxyethane (DME) and 1,3-dioxolane (DOL) (1:1), with a density of ∼1.2 g ml^−1^ (Liu T. et al., 2021). Although a maximum electrolyte amount of 11 μl mg_s_^−1^ (E/C = 7 µl mAh^−1^) was defined to surpass the specific energy of Li-ion batteries at full sulfur utilization, many experimental reports established that lean electrolyte conditions of <5 μl mg_s_^−1^ (E/C = ∼3 µl mAh^−1^) were essential to obtain high-energy-density Li-S batteries (Hagen et al., 2015; McCloskey, 2015; Chen J. et al., 2017; Zhao et al., 2020a; Dörfler et al., 2020; Liu T. et al., 2021). Consequently, many studies are mainly focused on lowering the electrolyte volume to reduce its weight fraction and, hence, improve the energy density of the Li-S battery. However, the density of the liquid electrolyte should be also considered since low-density electrolytes will have a greater impact on the delivered energy density at the same E/C ratio.

Here, we report a systematic analysis of Li-S pouch cells based on a standard sulfur cathode to understand the impact that ASL and electrolyte amount have on the delivered energy density. Multilayered pouch cells in this work comprise cathodes with different ASL (2.0, 4.0, and 5.3 mg_s_ cm^−2^), low N/P ratios (1.6 < N/*p* < 2.3), lean electrolyte conditions with a low ratio of electrolyte-volume to cell capacity (E/C ratio = 3.5 and 3.0 µl mAh^−1^), and a low-density electrolyte (∼1.0 g ml^−1^), stating the importance of each battery component in terms of their weight fraction within the whole battery. Additionally, the effect of the high loading of sulfur cathodes and the amount of the electrolyte on power density and the reversibility of the system were analyzed. The obtained electrochemical results demonstrate a remarkable improvement in the energy density (436 Wh kg^−1^) when reducing the electrolyte density and E/C ratio down to 3.0 µl mAh^−1^, and, subsequently, increasing the ASL up to 5.3 mg_s_ cm^−2^.

## Materials and Methods

### Cathode Fabrication

The cathodes were fabricated using 66 wt% commercial sulfur powder (<40 µm Merck). A mixture of sulfur (S) and conductive additive (carbon black, TIMCAL C-NERGY SUPER C45, Imerys Graphite, and Carbon) were added to a solution of polyvinylidene fluoride (PVDF) 5,130 (SOLVAY) in *N*-methylpyrrolidone (NMP) to form a cathodic slurry. The slurry was then blade casted onto the carbon-coated aluminum foil (MTI Corp) and dried in a dry room (dew point −50°C) at 60°C under a dynamic vacuum for 12 h before cell assembly.

### Electrochemical Characterization

The prepared cathodes were used for the assembly of Li–S pouch cells. The cathodes were prepared with different sulfur loadings (2.0, 4.0, and 5.3 mg_s_ cm^−2^) and different theoretical capacities (3.3, 6.6, and 8.9 mAh cm^−2^, respectively), derived from the theoretical capacity of elemental sulfur (1,672 mAh g^−1^).

One layer of a commercial polyolefin separator, Celgard 2,500 was used. The electrolyte was based on 0.38 M LiTFSI (Sigma Aldrich) and 0.32 M LiNO_3_ (Sigma Aldrich) as an additive in a 3/1 (v/v) mixture of DME and DOL (both purchased from BASF). Lithium foils with a thickness of 50, 75, 100, and 125 µm purchased from Rockwood Lithium were used as the anode.

Vacuum drying of electrodes and cell assembly was conducted in a dry room with a dew point below −50°C. Thereafter, the assembled pouch cells were cycled in a BaSyTec Cell Test System (Germany) at 25 ± 1°C controlled by air conditioning.

The electrochemical behavior of the assembled pouch cell was evaluated at different discharge C-rates (C/20, C/10, C/5, C/2.5, and 1C), considering the theoretical capacity of elemental sulfur (C = 1,672 mAh g^−1^). The cycle life of the pouch cells was investigated within a 1.9–2.5 V cycling interval at C/10 charge-discharge current C-rate.

## Results

**FIGURE 3:**
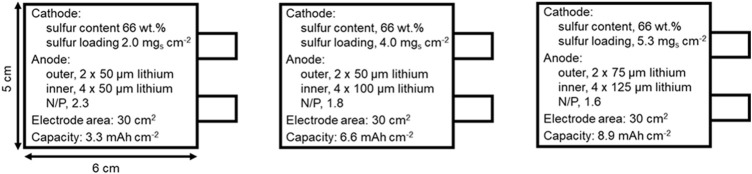
Design parameters of the fabricated Li-S pouch cells.

Due to the well-known limitations of the conducted studies at the coin cell level, we decided to investigate the impact of different battery components, especially, cathode and electrolyte according to their contribution to the cell performance (Chung and Manthiram, 2018; Chen et al., 2019b). Li–S pouch cells were designed and assembled with five double-side coated cathodes and six lithium metal foil anodes, with an electrode width of 5 cm and a length of 6 cm (Figure 3; Supplementary Table S1). The sulfur content of the cathode was 66 wt% with different ASL (2.0, 4.0, and 5.3 mg_s_ cm^−2^) to render different areal capacities (3.3, 6.6, and 8.9 mAh cm^−2^, respectively) according to the theoretical specific capacity of sulfur (1,672 mAh g^−1^). It is worth noting that due to the standard fabrication method of the cathode (where a commercial sulfur powder was directly mixed with a conductive carbon additive and a binder solution); we found that an ASL threshold is 5.3 mg_s_ cm^−2^. We should also clarify that commercial lithium metal foils with a thickness of 50, 75, 100, and 125 µm were used to assemble Li-S pouch cells in the attempt of providing an adequate balance between the cathode and the anode (i.e. N/P). Despite aiming N/*p* = 2, different N/P ratios were induced for each ASL, due to the limited availability of thickness variety of commercial Li metal foil. Moreover, considering the typically-used ether-based liquid electrolytes for Li-S batteries, the concentration of LiTFSI and LiNO_3_ salts was reduced to a final concentration of 0.38 and 0.32 M, respectively, lowering the density of the electrolyte (∼1.0 g ml^−1^), which reduced its mass contribution to the cell.

**FIGURE 4:**
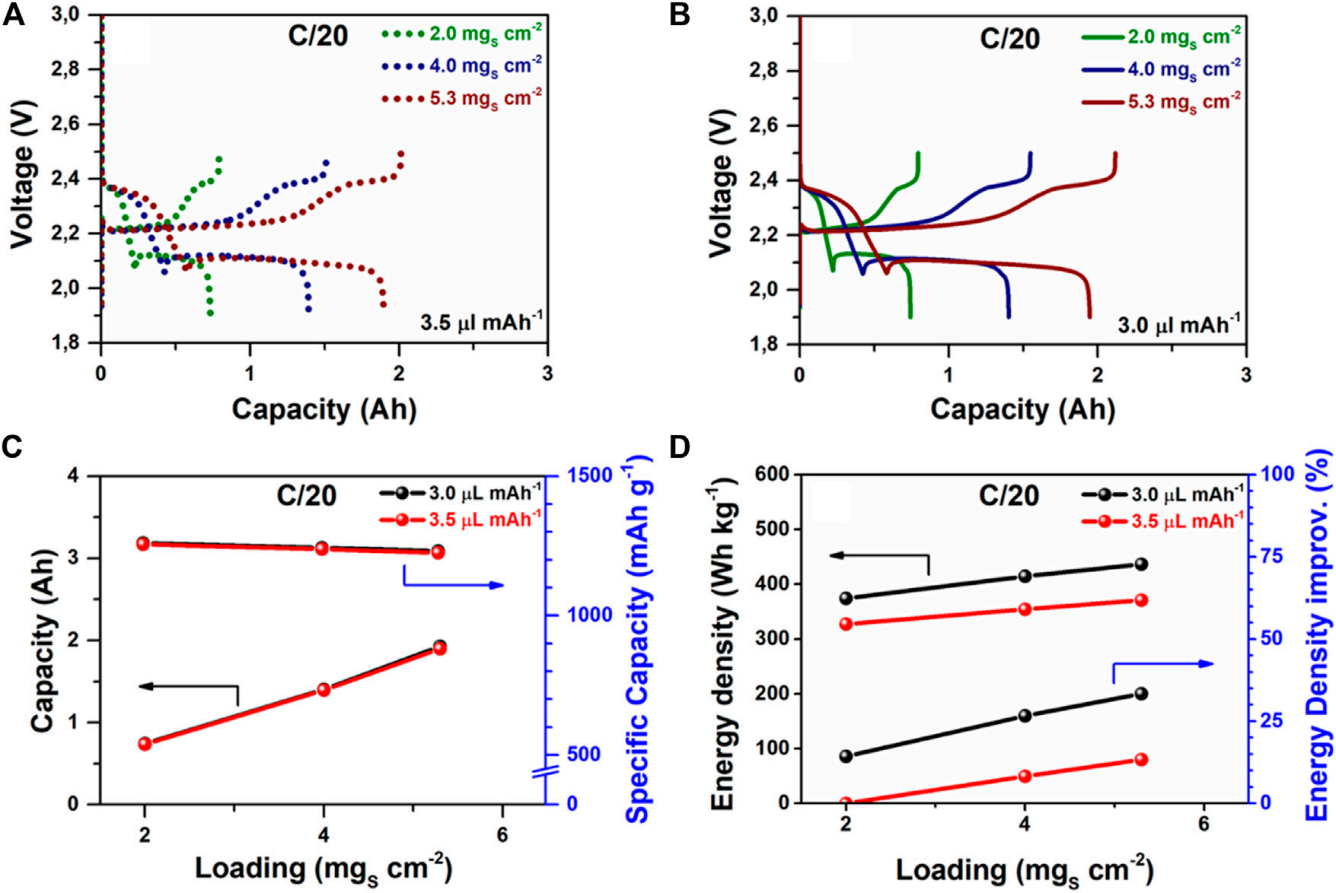
Electrochemical performance of Li-S pouch-cells with different ASL (2.0, 4.0, and 5.3 mg_s_ cm^−2^) and at different E/C ratios (3.0 and 3.5 µl mAh^−1^) at a current density of C/20. Galvanostatic charge/discharge profiles of Li-S batteries with **(A)** 3.5 µl mAh^−1^ and **(B)** 3.0 µl mAh^−1^. **(C)** Capacity and specific capacity per sulfur mass (mAh g_s_^−1^g^−1^_sulfur_^.^) and **(D)** energy density and corresponding energy density improvement of Li-S pouch-cells (reference case: low ASL = 2.0 mg_s_ cm^−2^ and E/C ratio = 3.5 µl mAh^−1^).

In total, six Li-S pouch cells were fabricated, where each ASL was tested with different E/C ratios (3.5 and 3.0 µl mAh^−1^) to study: 1) the contribution of each cell component to the overall specific energy of the battery; 2) the impact of the cathode ASL and the E/C ratio on the electrochemical performance of the Li-S system at different current densities, and 3) the impact of the cathode ASL and the E/C ratio on the cyclability of the system. In terms of cell capacity, at low current densities (C/20), slight differences were observed on decreasing the E/C ratio from 3.5 to 3.0 µl mAh^−1^, which represented an electrolyte reduction of ∼17 wt%. Figures 4A,B depict the voltage profiles of Li-S pouch-cells with different ASL cycled at a current density of C/20. Pouch cells based on cathodes with an ASL of 2.0 mg_s_ cm^−2^ delivered 0.73 Ah (∼1,260 mAh g^−1^) and 0.74 Ah (∼1,225 mAh g^−1^) for an E/C ratio of 3.0 and 3.5 µl mAh^−1^, respectively. As it was expected, the cathodes with 4.0 mg_s_ cm^−2^ and 5.3 mg_s_ cm^−2^ proportionally increased the cell capacities according to their ASL, by delivering ∼1.5 Ah and ∼1.9 Ah, respectively at both E/C ratios (Figure 4C). These results evinced that, under lean electrolyte conditions and low current densities, investigated Li-S pouch cells showed no capacity losses at low, medium, or high ASL.

**FIGURE 5:**
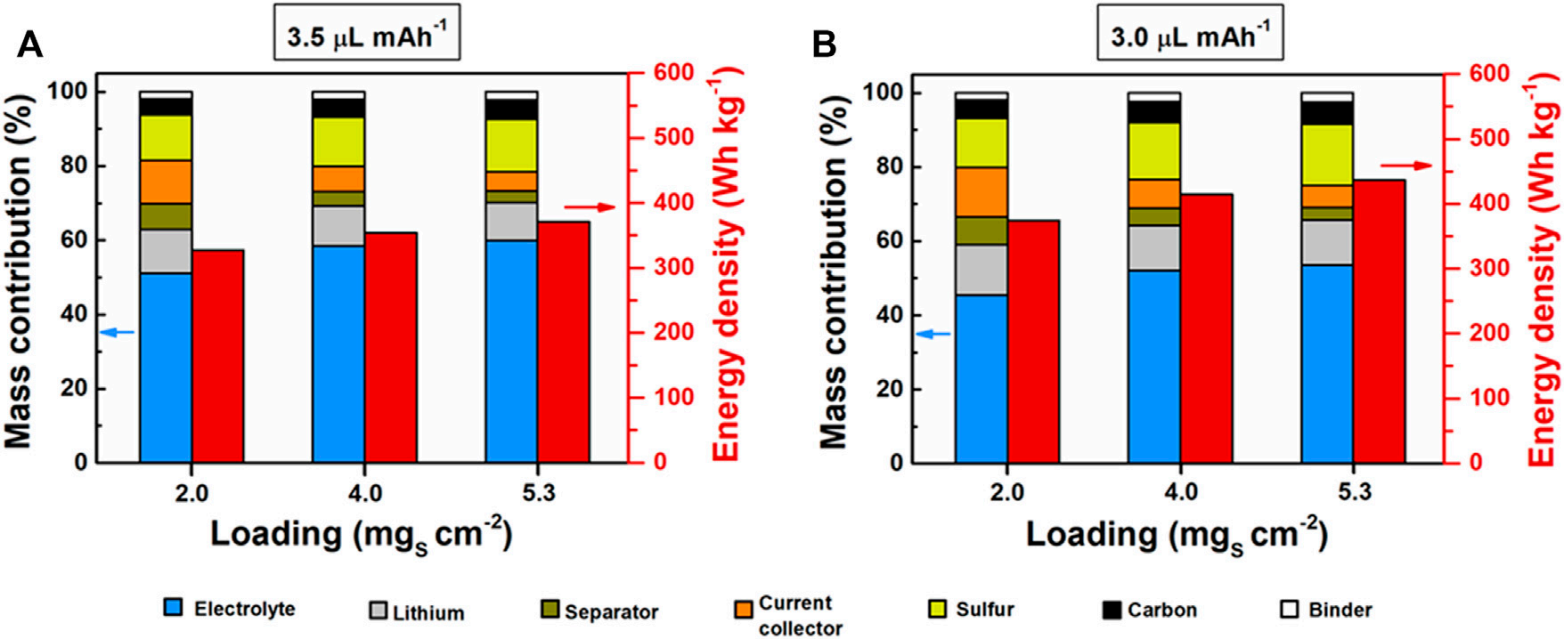
Mass distribution and corresponding energy density of fabricated Li-S pouch cells with **(A)** E/C ratio = 3.5 µl mAh^−1^ and **(B)** E/C ratio = 3.0 µl mAh^−1^. More detailed information can be found in Supplementary Tables S1, S2 of Supporting Information.

On the other hand, considering the total weight of each cell (excluding tabs and packaging), the mass reduction of the electrolyte in Li-S pouch cells was analyzed from the point of view of the delivered specific discharge energy measured at C/20. While Figure 4D shows the specific energy values, Figures 5A,B provides an overview of the specific energy trend according to the mass contribution of each pouch-cell component. At a fixed E/C ratio of 3.5 µl mAh^−1^ (Figures 4D, 5A), the mass contribution of sulfur and electrolyte in each pouch cell was increased by ∼17 wt% when modifying the ASL from 2.0 mg_s_ cm^−2^ (12.2 wt% sulfur; 51.0 wt% electrolyte) to 5.3 mg_s_ cm^−2^ (14.2 wt% sulfur; 59.9 wt% electrolyte). It should be noted that by increasing the ASL higher, the weight fractions of sulfur and the electrolyte caused the decrease in the relative weight of the inactive components, such as the separator and the aluminum current collector. This effect moderately boosted the delivered specific energy from 327 Wh kg^−1^ to 371 Wh kg^−1^, representing an improvement of ∼13 wt% at an E/C ratio of 3.5 µl mAh^−1^. All the details for each E/C ratio and ASL are included in Supplementary Tables S2, S3 in Supporting Information, demonstrating that there were no significant variations in the delivered specific energies.

Low ASL and high electrolyte volume are the typical conditions for Li-S batteries in the coin-cell format (Chen et al., 2019a). Therefore, the specific energy (327 Wh kg^−1^) provided by a Li-S pouch cell with a low ASL of 2.0 mg_s_ cm^−2^ and an E/C ratio of 3.5 µl mAh^−1^ will be used as a reference system (RS) for further analysis. In general, the reduction of the E/C ratio from 3.5 µl mAh^−1^ to 3.0 µl mAh^−1^ caused a strong effect on the improvement of the cell-specific energy. Interestingly, even at low ASL (2.0 mg_s_ cm^−2^), a similar specific energy value (374 Wh kg^−1^) could be obtained by decreasing the E/C ratio to 3.0 µl mAh^−1^, in comparison with the Li-S system based on high ASL (5.3 mg_s_ cm^−2^) and E/C ratio of 3.5 µl mAh^−1^ (371 Wh kg^−1^), stating the importance of the electrolyte amount in the delivered specific energy of Li-S batteries (Figures 4D, 5B). Figure 5B demonstrates that it is possible to boost the specific energy of the system up to 436 Wh kg^−1^ through the simultaneous reduction of the E/C ratio to 3.0 µl mAh^−1^ and ASL increase to 5.3 mg_s_ cm^−2^. This represented an improvement in the specific energy of the system of ∼33% in comparison with the RS, as shown in Figure 4D.

In summary, it has been demonstrated that it is possible to reach a high energy density (>400 Wh kg^−1^) for Li-S pouch cells with high-sulfur-loaded cathodes (5.3 mg_S_ cm^−2^), low N/P ratios (1.6 < N/*p* < 2.3), and a reduced E/C ratio (3.0 µl mAh^1^), which are the most demanded parameters for practical high-energy density Li-S batteries (Chung and Manthiram, 2018; Zhu et al., 2019; Dörfler et al., 2020; Ye et al., 2022).

**FIGURE 6:**
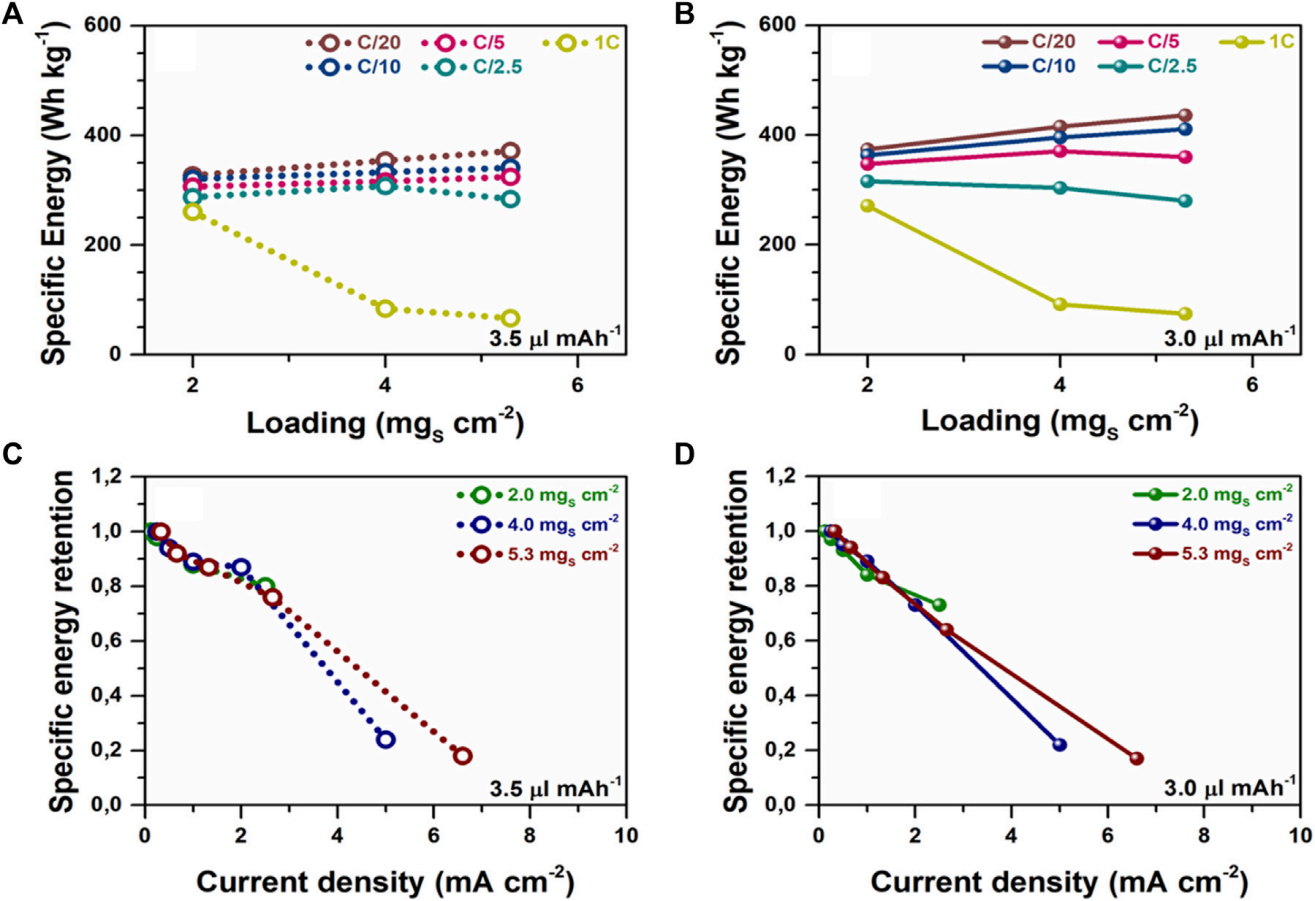
Discharge C-rate performance of Li-S pouch-cells with different ASL (2.0, 4.0, and 5.3 mg_s_ cm^−2^): Specific energy densities of cells with **(A)** E/C 3.5 µl mAh^−1^ and **(B)** E/C 3.0 µl mAh^−1^. Discharge energy retention versus current density of cells with **(C)** E/C 3.5 µl mAh^−1^ **(D)** and E/C 3.0 µl mAh^−1^. NOTE: Tabs and packaging were excluded from the specific energy calculations.

The results of further electrochemical studies presented in Figures 6A,B show charge-discharge C-rate performance of Li-S pouch cells with different ASL (2.0, 4.0, and 5.3 mg_s_ cm^−2^) at C/20, C/10, C/5, C/2.5, and 1C discharge current densities. These experimental results show that, for a fixed ASL, all tested cells with an E/C ratio = 3.0 µl mAh^−1^ presented superior specific energies, not only at low current densities (C/20) but also at moderate (C/2.5) or even high (1C) current densities, comparing to cells with an E/C ratio of 3.5 µl mAh^−1^. However, under lean electrolyte conditions, reducing the E/C ratio from 3.5 µl mAh^−1^ to 3.0 µl mAh^−1^ led to lower energy retentions, for each ASL, when comparing the delivered specific energies at low (C/20) and medium (C/2.5) current densities. For example, Li-S pouch cells with a low ASL of 2.0 mg_s_ cm^−2^ exhibited retentions of ∼88% (E/C = 3.5 µl mAh^−1^) and ∼84% (E/C = 3.0 µl mAh^−1^) between medium and low current densities, while cells with a high ASL of 5.3 mg_s_ cm^−2^ were able to retain 76 and 64%, respectively. These results manifest the key role of the electrolyte volume on the delivered specific energy values at medium current densities. Additionally, a common electrochemical behavior was detected in all tested Li-S pouch cells. At low current densities (C/20 or C/10), the specific energy was improved according to the increase in cathode ASL. At the same time, at medium (C/5 and C/2.5) and, especially, at high current densities (1C) the specific energies were lowered at higher cathode ASL. In fact, it was interesting to observe that this effect was even clearer in cells with higher ASL and lower E/C ratios.

Although it is possible to find extensive literature which reported Li-S batteries cycling at high rates (1C, 2C, or even 5C), these experimental results were obtained using the coin cell format with a large excess of electrolyte and high N/P ratio (Qiu et al., 2014; Chabu et al., 2017; Zheng et al., 2017). Under these conditions, it is reported that the main limitation of the systems at high current densities is related to the slow kinetics of the sulfur cathode and poor charge transport (Robinson et al., 2021). Therefore, for the improvement of the C-rate performance, many approaches in the literature are focused on the development of novel sulfur hosting materials, with both highly-conductive and electrocatalytic properties, to mediate the polysulfide conversion reactions (Eftekhari and Kim, 2017; Liu et al., 2018).

At the same time, in this study, the results obtained at the pouch cell level under lean electrolyte conditions showed that despite that all the cells presented the same E/C ratio and similar excess of Li metal anode capacity, the behavior of the system was mainly affected by the ASL of the cathode. Therefore, in order to clarify this phenomenon, experimental data of Figures 6A,B was re-plotted to depict the specific energy retention versus the current density (Figures 6C,D). Although it is well-known that the electrochemical performance of Li-S batteries significantly differs depending on the cathode ASL, a severe decrease in the specific energy retention upon discharge C-rate increasing was observed for all tested cells when the current density exceeded 1.5–2.0 mA cm^2^, at a fixed E/C ratio of 3.5 µl mAh^−1^ (Figure 6C). This effect was even more severe when the E/C ratio was reduced to 3.0 µl mAh^−1^ (Figure 6D). These results demonstrate that the specific energy retention of Li-S pouch cells is severely affected at high discharge current densities. These results are in agreement with previous studies on lithium metal batteries which suggest that at high areal current densities the C-rate capability limitation is caused not only by the sulfur cathode but also by the electrolyte phase within the porous electrode structure, rather than in the separator or the Li-metal electrode (Wu et al., 2012).

Therefore, a better understanding of the electrolyte transport properties in the porous electrode structure is required to accurately explain the energy density fade at high discharge currents. We consider that this finding paves the way for the further improvement of the power density of the Li-S system.

**FIGURE 7:**
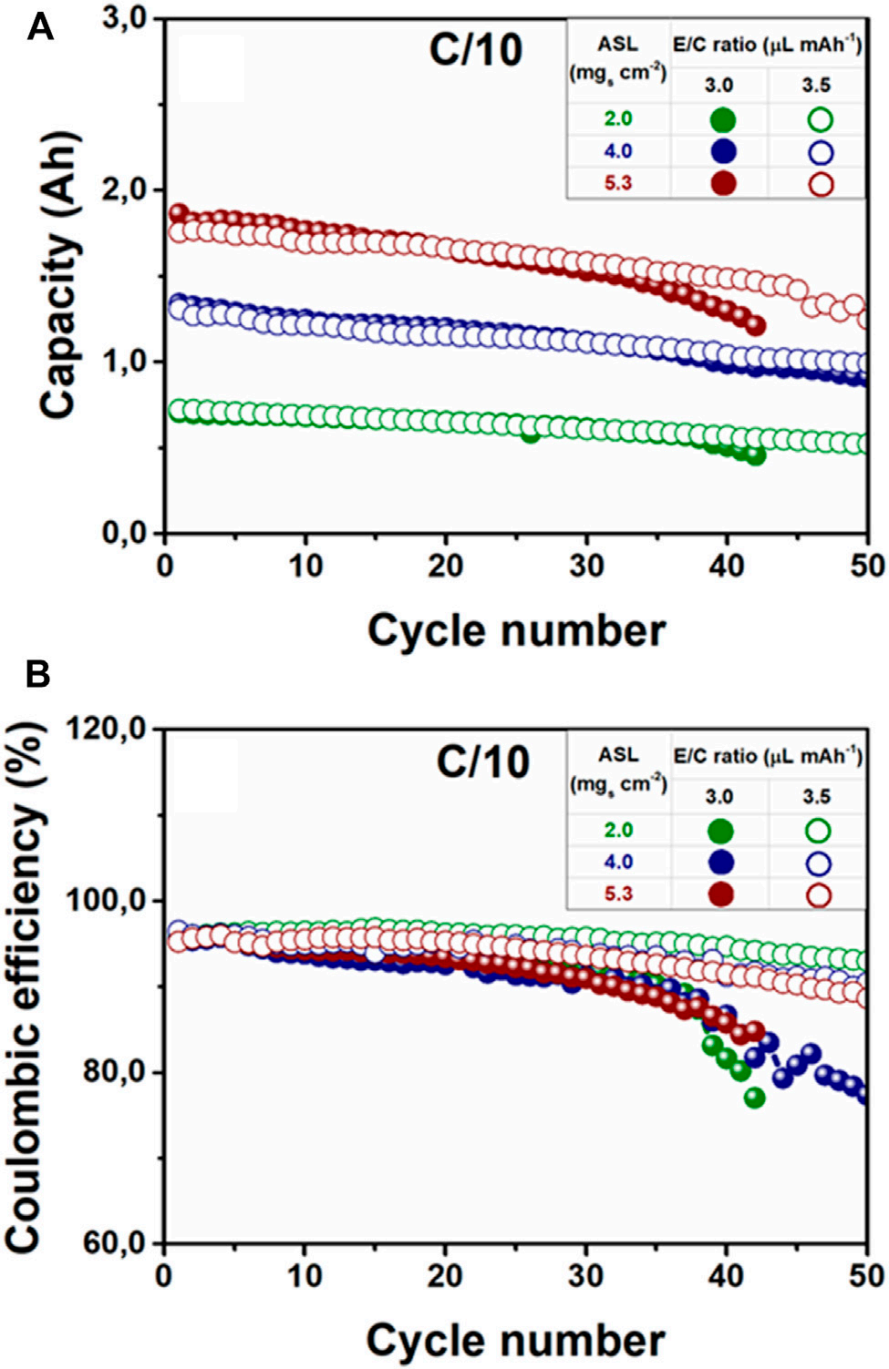
**(A)** Galvanostatic long cycling performance and **(B)** Coulombic efficiency of Li-S batteries with different ASL (2.0, 4.0, and 5.3 mg_s_ cm^−2^) and different E/C ratios (3.0 and 3.5 µl mAh^−1^).

Moreover, the long-term electrochemical performance of tested Li-S pouch cells revealed additional limitations, since galvanostatic cycling tests evinced the existence of a common failure mechanism of the tested pouch cells (Figures 7A,B; Supplementary Figures S1A–F). Li-S pouch-cells with an E/C ratio of 3.0 µl mAh^−1^ exhibited a notorious capacity loss and a severe Coulombic efficiency fading upon continuous cycling (>40 cycles). It should be noted that in the literature this phenomenon is typically attributed to the cathode in coin-cell Li-S batteries, whose cycling stability is mainly dominated by the shuttle effect of LiPS. In addition to that, electrolyte and lithium are used in excess in Li-S coin cells, which discards the fast degradation of the Li metal anode. According to some researchers, low capacities and inferior cyclability in Li-S pouch-cells are due to cathode-electrolyte interfacial processes, which induce mass transfer limitations because of LiPSs accumulation, which has been already demonstrated (Zhao et al., 2021). However, it is also known that continuous cycling in Li-S pouch cells causes damage and degradation at the surface of the Li metal anode, since plating/stripping occurs randomly at high current densities, leading to the uneven deposition of lithium (Jiao et al., 2018; Kong et al., 2019). Consequently, the solid electrolyte interphase (SEI) formed at the Li metal anode becomes unstable and Li dendrite formation is enhanced. As a result, the anode results are irreversibly damaged and “dead” lithium is detached from the surface, exposing unreacted Li which further reacts with the remaining electrolyte. This reaction increases the consumption of the electrolyte and, eventually, leads to electrolyte depletion which provokes a fast end of the cell cycling life. Considering that this effect could be much aggravated under lean electrolyte conditions, the unstable cycling performance and low Coulombic efficiencies observed in Li-S pouch cells with a fixed E/C ratio of 3.0 µl mAh^−1^, in comparison with the counterparts (E/C ratio of 3.5 µl mAh^−1^), could be caused by the volume reduction of the electrolyte.

Additionally, the Li metal anode also exhibited its cycling performance limitation at an E/C ratio of 3.5 µl mAh^−1^ with a high ASL of 5.3 mg_s_ cm^−2^. Considering that the charge current density was established depending on the sulfur loading, medium and high ASL (>4.0 mg_s_ cm^−2^) induced higher area current densities for a fixed C-rate. From the cathode point of view, it has been reported that high charge/discharge currents could lead to poor electrochemical performance. The low electronic conductivity of the sulfur electrodes, despite the high content of conductive-carbon additive, could lead to inhomogeneous charge-density distribution along the sulfur-based cathode and result in incomplete sulfur utilization (Robinson et al., 2021). However, according to recent literature, the electrochemical behavior of Li metal-based pouch cells at high current densities could be also limited by the Li metal anode, since the capacity retention in Li metal-based pouch cells has been reported to be severely affected when applying high current densities (Cheng et al., 2017, 2018; Jiao et al., 2018). For example, Jiao et al. performed a thorough characterization of the Li metal anode in a pouch cell battery (Jiao et al., 2018). At low charge current densities (0.8 mA cm^−2^), the Li metal anode exhibited moderate corrosion and low cracking at the surface, due to low Li utilization. In contrast, medium (1.3 mA cm^−2^) and high current densities (2.0 mA cm^−2^) demanded higher anode utilization, which induced severe corrosion and degradation, transforming the Li metal anode into a porous structure that led to the cell failure. Therefore, the long cycling results obtained for our highly-loaded Li-S pouch cells are in good agreement with the literature, since higher current densities could eventually damage the Li metal anode, thus affecting the cycling performance of the cell. In fact, the field emission scanning electron microscopy (FE-SEM) images in Supplementary Figure S2 provided solid evidence to confirm the obtained electrochemical results. Considering the leanest electrolyte conditions in our tested Li-S pouch cells (E/C = 3 0.0 µl mAh^−1^), it was possible to observe the gradual degradation of the Li-metal surface according to the areal sulfur loading of the cathode. Low areal sulfur loadings (2.0 mg_s_ cm^−2^) were tested at low charge current densities, which induced the formation of mossy lithium and a moderate degradation of the Li-metal anode surface, affecting its electrochemical performance. Added to that, medium and high areal sulfur loadings (4.0 and 5.3 mg_s_ cm^−2^) required higher charge current densities for a fixed C-rate of the cell, which severely damaged the surface of the analyzed Li-metal anodes by inducing the formation of lithium dendrites and, even, “dead” lithium. Consequently, it has been demonstrated that Li-S batteries, based on highly-loaded sulfur cathodes, and lithium metal anodes under lean electrolyte conditions, will require novel Li-metal anode strategies for the highly-demanding charge current densities established by the sulfur-cathode loading.

## Discussion

In this work, key aspects to understanding the performance of high-energy-density Li-S batteries have been evaluated. The electrochemical studies performed have manifested the relevance of sulfur loading and the impact of electrolyte volume reduction, over the delivered energy density of the Li-S pouch cell configuration. It has been demonstrated that it is possible to develop high-energy-density (>430 Wh kg^1^) Li-S pouch cells by reducing the mass contribution of inactive cell components by adequately balancing sulfur loading (5.3 mg_s_ cm^−2^) and electrolyte amount (3.0 µl mAh^−1^). Noteworthy, we strongly believe that enhanced energy densities could be achieved by further optimization of cell components.

From the cathode side, improving the processability of the sulfur cathode could allow increasing the ASL and therefore, the reduction of the weight contribution of the aluminum current collector and the separator. In addition to that, the calendering of porous electrodes is well-known to have a substantial effect on the electrode by reducing its porosity. Application of this compression method to the sulfur-based cathode could have a great impact on the balancing of the electrolyte. Less porosity would minimize the volume of electrolyte required to fully wet the sulfur-based cathode, which could yield extremely low E/C ratios, improved sulfur utilization, and, therefore, higher energy densities. From the electrolyte point of view, it could be also interesting to study alternative systems with lower densities when compared with the standard electrolyte solution (<1.2 g ml^−1^). Reducing the salt concentration or selecting solvents with low density would directly decrease the weight contribution of the electrolyte and therefore increase the delivered energy density of the Li-S battery.

Nevertheless, some limitations have been observed. Li metal anodes have been claimed as the main cellular component responsible for the system failure when cycling at high current densities. According to previous reports, high charge/discharge current densities induce the formation of mossy Li, which constantly and irreversibly consumes the electrolyte, further leading to electrolyte depletion. We have observed that this effect is aggravated when reducing the E/C ratio since our experimental results with a lower E/C ratio (3.0 µl mAh^−1^) exhibited a more pronounced irreversible capacity fading upon continuous cycling. Therefore, the commercialization of the high-energy-density Li-S batteries will demand improvement in the properties of the Li metal anode and any attempted strategy should confer high robustness to the Li metal anode under lean electrolyte conditions. These strategies, for example, *in situ*/*ex situ* anode protection or the development of scalable three-dimensional lithium metal anodes, should minimize and/or postpone the formation of mossy Li and the further degradation of the Li metal anode, which would improve the electrochemical stability and ensure extended cycling life for high-energy-density Li-S cells. It is also worth mentioning that, to avoid the issues derived from using lithium metal anodes, other approaches propose lithium-free Li-S batteries. For example, some studies suggest the implementation of the Li_2_S-based cathodes as prelithiation agents for silicon or graphite anodes in Li-S batteries (Xing et al., 2020). Nevertheless, despite improving the electrochemical performance of the Li-S batteries, due to the introduction of transition metals (e.g. W, Mo, Ti, and Co, etc.) to increase the electronic/ionic conductivity and provide a strong interaction between the corresponding transition metal and LiPSs, the impact of this strategy should be studied in the practical energy density delivered by the Li-S battery.

## Data Availability

The original contributions presented in the study are included in the article/Supplementary Material, further inquiries can be directed to the corresponding author.
