# Supplementary material for: Understanding of Crucial Factors for Improving the Energy Density of Lithium-Sulfur Pouch Cells
Source: Front Chem. 2022 May 2;10:888750. doi: 10.3389/fchem.2022.888750 (PMC9108244; doi:10.3389/fchem.2022.888750)
Supplement: Supplementary file 1 [file DataSheet1.docx]

Supplementary Material

Table S1. Parameters of designed Li-S pouch cells.

| **Theoretical areal capacity (mAh cm^-2^)** |  | 3.3 | 6.6 | 8.9 | 3.3 | 6.6 | 8.9 |  |
| --- | --- | --- | --- | --- | --- | --- | --- | --- |
| **Cell dimensions** |  | 6.0 cm x 5.0 cm | 6.0 cm x 5.0 cm | 6.0 cm x 5.0 cm | 6.0 cm x 5.0 cm | 6.0 cm x 5.0 cm | 6.0 cm x 5.0 cm |  |
|  |  |  |  |  |  |  |  |  |
| **Structure** | Li metal anode foil | 6 | 4 (inner)  2 (outer) | 4 (inner) 2 (outer) | 6 | 4 (inner) 2 (outer) | 4 (inner) 2 (outer) |  |
|  | Double-sided S-C cathode | 5 | 5 | 5 | 5 | 5 | 5 |  |
| **Cathode** | Sulfur/carbon/binder ratio | 66/24/10 | 66/24/10 | 66/24/10 | 66/24/10 | 66/24/10 | 66/24/10 |  |
|  | Sulfur areal loading (mg cm^-2^) | 2.0 | 4.0 | 5.3 | 2.0 | 4.0 | 5.3 |  |
|  | Sulfur specific capacity @C/20 (mAh g^-1^) | 1260 | 1243 | 1231 | 1255 | 1238 | 1225 |  |
| **Electrolyte** | 0.38 M LiTFSI / 0.32 LiNO_3_ DOL/DME (1/3, v/v) | 1.998 g E/S ⁓3.5 µL mg^-1^ E/C ⁓3.0 µL mAh^-1^ | 3.996 g E/S ⁓3.6 µL mg^-1^ E/C ⁓3.0 µL mAh^-1^ | 5.335 g E/S ⁓3.4 µL mg^-1^ E/C ⁓3.0 µL mAh^-1^ | 2.498 g E/S ⁓ 4.2 µL mg^-1^ E/C ⁓3.5 µL mAh^-1^ | 4.995 g E/S ⁓4.4 µL mg^-1^ E/C ⁓3.5 µL mAh^-1^ | 6.693 g E/S ⁓4.3 µL mg^-1^ E/C ⁓3.5 µL mAh^-1^ |  |
|  |  |  |  |  |  |  |  |  |
|  |  |  |  |  |  |  |  |  |
|  |  |  |  |  |  |  |  |  |
| **Anode** | Li metal foil | 50 µm; N/P = 2.3 | 100 µm (inner); 50 µm (outer); N/P = 1.8 | 125 µm (inner); 75 µm (outer); N/P = 1.6 | 50 µm; N/P = 2.3 | 100 µm (inner); 50 µm (outer); N/P = 1.8 | 125 µm (inner); 75 µm (outer); N/P = 1.6 |  |

Table S2. Weight fraction contribution of each cell component in Li-S pouch cells with different ASL and a E/C ratio of 3.5 µL mAh^-1^.

| **3.5 µL·mAh^-1^** | | | | | | | | |
| --- | --- | --- | --- | --- | --- | --- | --- | --- |
| **Areal  sulfur loading  (mg_s_·cm^-2^)** | **Mass contribution (wt%)** | | | | | | | **Specific energy  (Wh·kg^-1^)** |
|  | **Electrolyte** | **Lithium** | **Separator** | **Current collector** | **Cathode** | | |  |
|  |  |  |  |  | **Sulfur** | **Carbon** | **Binder** |  |
| 2.0 | 51.1 | 11.9 | 6.9 | 11.7 | 12.2 | 4.4 | 1.8 | 327 |
| 4.0 | 58.5 | 10.8 | 3.9 | 6.7 | 13.3 | 4.8 | 2.0 | 354 |
| 5.3 | 59.9 | 10.3 | 3.0 | 5.2 | 14.3 | 5.2 | 2.2 | 371 |

Table S3. Weight fraction contribution of each cell component in Li-S pouch cells with different ASL and a E/C ratio of 3.0 µL mAh^-1^.

| **3.0 µL mAh^-1^** | | | | | | | | |
| --- | --- | --- | --- | --- | --- | --- | --- | --- |
| **Areal  sulfur loading  (mg_s_ cm^-2^)** | **Mass contribution (wt%)** | | | | | | | **Specific energy  (Wh kg^-1^)** |
|  | **Electrolyte** | **Lithium** | **Separator** | **Current collector** | **Cathode** | | |  |
|  |  |  |  |  | **Sulfur** | **Carbon** | **Binder** |  |
| 2.0 | 45.4 | 13.5 | 7.7 | 13.2 | 13.3 | 4.8 | 2.0 | 374 |
| 4.0 | 52.1 | 12.2 | 4.6 | 7.8 | 15.4 | 5.6 | 2.3 | 415 |
| 5.3 | 53.6 | 12.0 | 3.5 | 5.9 | 16.5 | 6.0 | 2.5 | 437 |


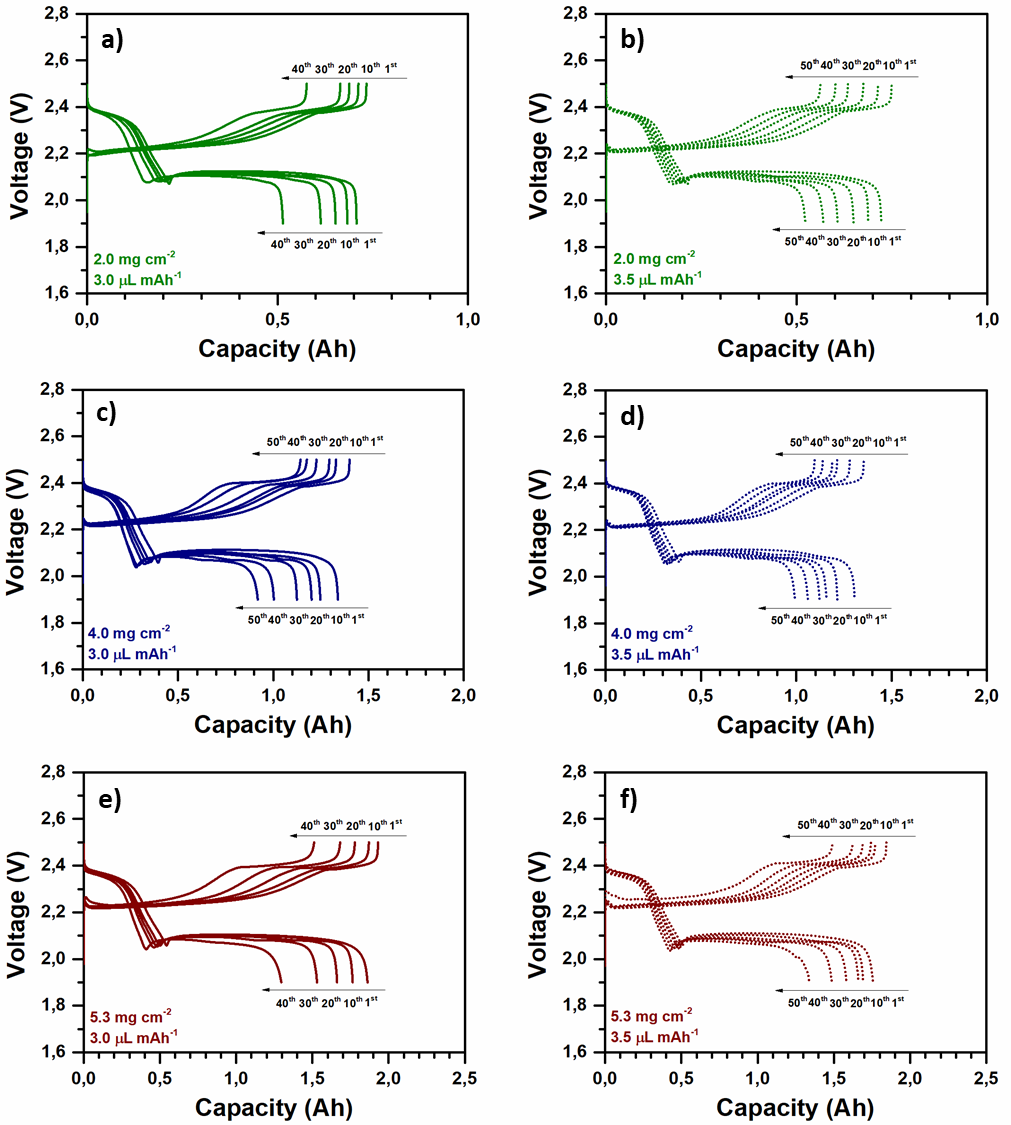


Figure S1. Galvanostatic charge/discharge profiles of Li-S batteries of Li-S pouch cells with different areal sulfur loadings (2.0, 4.0 and 5.3 mg_s_ cm^-2^) with a E/C ratio of (a, c, e,) 3.0 µL mAh^-1^ and (b, d, f) 3.5 µL mAh^‑1^.


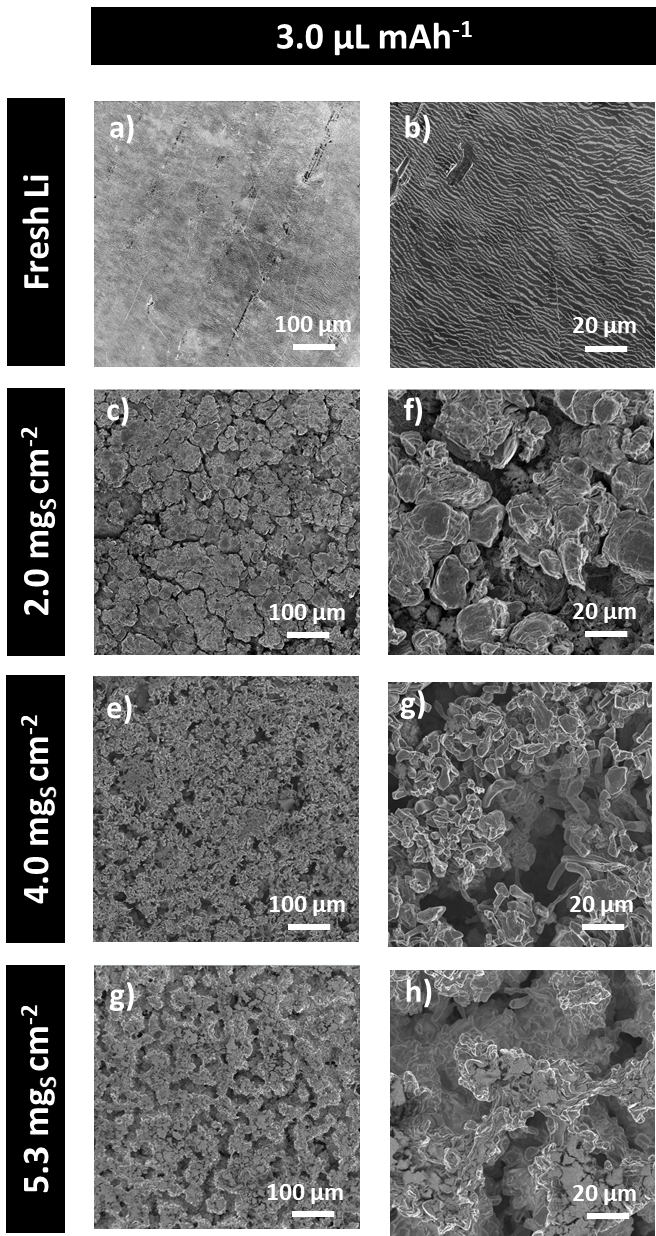


**Figure S2.** SEM images of (a,b) pristine lithium foil. Post-mortem SEM images of (c-h) lithium metal anodes cycled versus sulfur-based cathodes with different areal sulfur loadings (2.0, 4.0 and 5.3 mg_s_ cm^-2^) under lean electrolyte conditions (E/C = 3.0 µL mAh^-1^).
